# Supplementary material for: Tectal-derived interneurons contribute to phasic and tonic inhibition in the visual thalamus
Source: Nat Commun. 2016 Dec 8;7:13579. doi: 10.1038/ncomms13579 (PMC5155147; doi:10.1038/ncomms13579)
Supplement: Supplementary Information — Supplementary Figure 1. [file ncomms13579-s1.pdf]

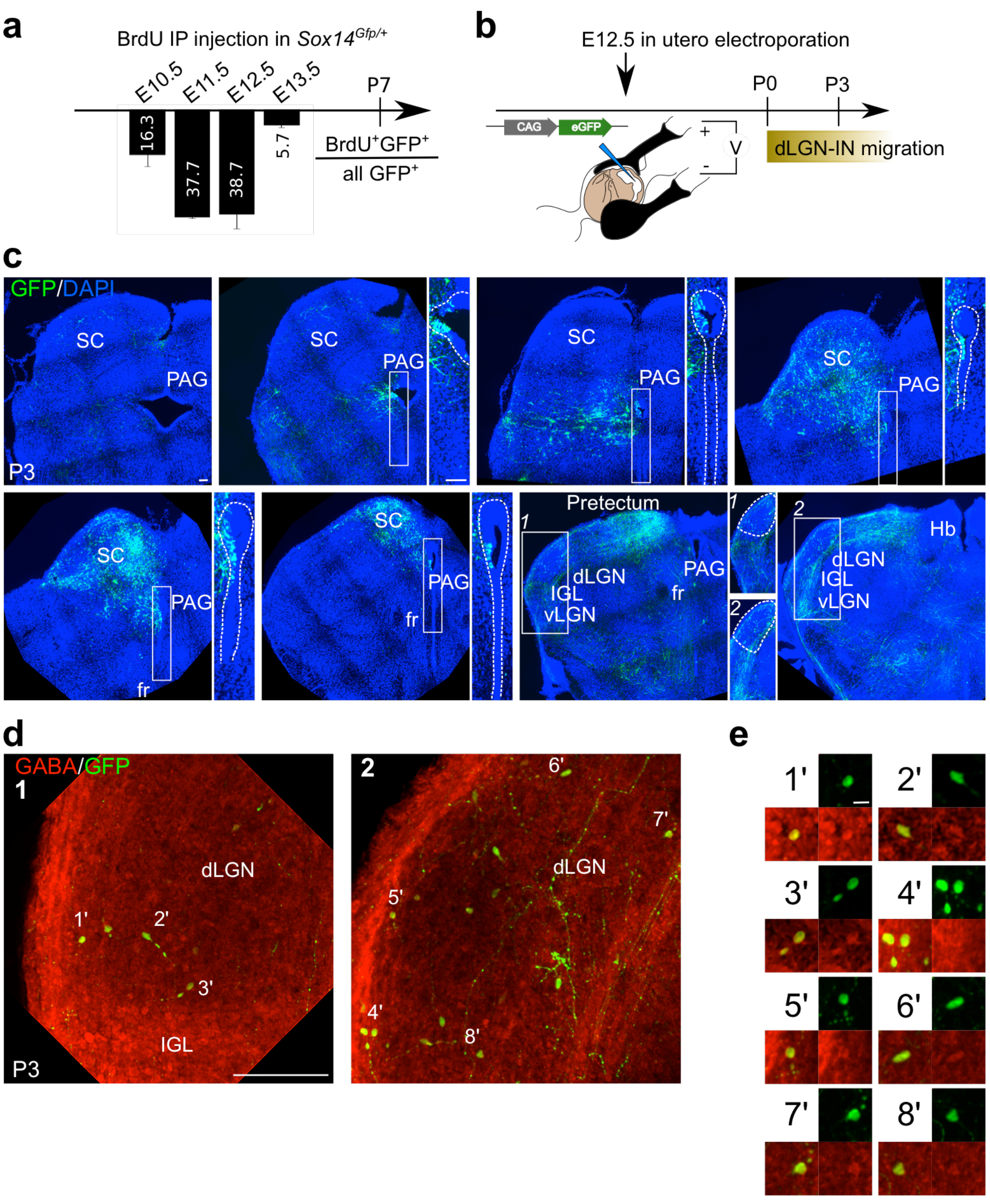

Supplementary Figure 1. Birth dating and fate mapping of dorsal midbrain progenitors. (a) BrdU incorporation in the genome of S-phase mitotic cells at embryonic days E10.5-E13.5 in *Sox14*$^{Gfp/+}$ embryos. Immunodetection of BrdU and GFP defines a peak of dLGN-IN genesis between E10.5 and E13.5. Close to 80% of dLGN-INs are born between E11.5 and E12.5 (fraction of GFP^{+} cells that are also BrdU^{+}: E10.5: 16.3 ± 4.2; E11.5: 38.7 ± 0.3; E12.5: 37.7 ± 5.2; E13.5: 5.7 ± 0.9. N=3 brains per time point; %, mean ± standard error). **(b)** Schematic illustration of *in utero* labelling of dorsal midbrain progenitors. Brains of wild type embryos were electroporated at E12.5 with a constitutive eGFP expression plasmid injected in the ventricular space of the midbrain, using asymmetric electrodes to restrict the labelled area to the rostral part of the dorsal midbrain. Analysis was carried out after birth (P3) to account for the postnatal migration of interneurons into the dLGN. **(c)** Electroporated brains were analysed first to establish the extent of the electroporated area by detection of GFP in the ependymal cells lining the brain ventricles, in serial sections along the caudo-rostral axis. Labelled ependymal cells are found clustered in a restricted area of the midbrain aqueduct, surrounded by the PAG. Differentiated GFP^{+} cells are largely contained within the SC and axonal projections are seen extending towards the pretectum and thalamus. More detailed analysis of the thalamus reveals an enrichment of GFP^{+} somas in the dLGN (insets *1* and *2*), although cells were also found in the vLGN and in the remaining thalamus in the following proportions - dLGN: 50%, vLGN/IGL: 11%, lateral posterior thalamic nucleus: 34% and ventrobasal thalamus: 5%, n = 427 cells from 1 brain. A similar distribution of labelled differentiated cells was observed in the thalamic nuclei when targeting dorsal midbrain progenitors in *Sox14*$^{cre/+}$ embryos at E12.5. The embryos were electroporated using a cre-dependent RFP expression plasmid together with the constitutive eGFP plasmid (data not shown). Scale bars are 100 μm. **(d)** Immunodetection of GABA within the dLGN confirms the inhibitory identity of the GFP-labelled cells. Scale bar is 100 μm. **(e)** Higher magnification of GFP-labelled cells in the dLGN showing the presence of GABA (white arrowheads). Scale bar is 10 μm. PAG: periaqueductal gray; SC: superior colliculus; fr: fasciculus retroflexus; Hb: habenular complex.
